# RecG and UvsW catalyse robust DNA rewinding critical for stalled DNA replication fork rescue

**DOI:** 10.1038/ncomms3368

**Published:** 2013-09-09

**Authors:** Maria Manosas, Senthil K. Perumal, Piero Bianco, Felix Ritort, Stephen J. Benkovic, Vincent Croquette

**Affiliations:** 1 Departament de Física Fonamental, Facultat de Física, Universitat de Barcelona, Diagonal 647, 08028 Barcelona, Spain; 2 CIBER-BBN de Bioingenieria, Biomateriales y Nanomedicina, Instituto de Sanidad Carlos III, Madrid, Spain; 3 Department of Chemistry, The Pennsylvania State University, University Park, 16802 Pennsylvania, USA; 4 Department of Microbiology and Immunology, Center for Single Molecule Biophysics, University at Buffalo, Buffalo, 14214, New York USA; 5 Laboratoire de Physique Statistique, Ecole Normale Supérieure, UPMC Univ. Paris 06, Université Paris Diderot, CNRS, 24 rue Lhomond, 75005 Paris, France; 6 Département de Biologie, Ecole Normale Supérieure, 46 rue d'Ulm, 75005 Paris, France

## Abstract

Helicases that both unwind and rewind DNA have central roles in DNA repair and genetic recombination. In contrast to unwinding, DNA rewinding by helicases has proved difficult to characterize biochemically because of its thermodynamically downhill nature. Here we use single-molecule assays to mechanically destabilize a DNA molecule and follow, in real time, unwinding and rewinding by two DNA repair helicases, bacteriophage T4 UvsW and *Escherichia coli* RecG. We find that both enzymes are robust rewinding enzymes, which can work against opposing forces as large as 35 pN, revealing their active character. The generation of work during the rewinding reaction allows them to couple rewinding to DNA unwinding and/or protein displacement reactions central to the rescue of stalled DNA replication forks. The overall results support a general mechanism for monomeric rewinding enzymes.

## Abstract (web summary)

The helicases UvsW and RecG have both unwinding and rewinding activities and are involved in the rescue of stalled DNA replication forks. Here Manosas *et al*. use single-molecule techniques to characterize the rewinding activities of the two helicases, concluding that rewinding is actively catalysed.

## Graphical abstract

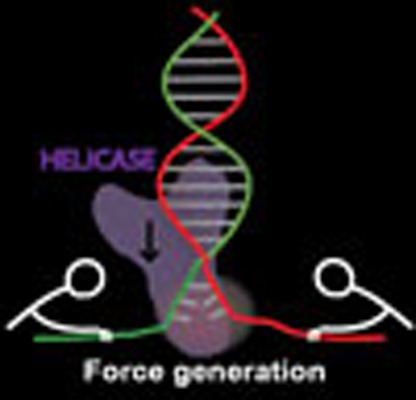

DNA helicases are involved in all aspects of DNA metabolism. These molecular motors use the energy of nucleoside triphosphate hydrolysis to move along DNA with a defined directionality (3′–5′ or 5′–3′)^1^,^2^. Many helicases use this unidirectional movement to promote the unwinding of duplex DNA^2^. In addition, some DNA-repair helicases are also capable of carrying out the reverse reaction, so-called DNA rewinding, facilitating the formation of duplex DNA from complementary single-stranded DNA (ssDNA)^3^,^4^,^5^,^6^,^7^,^8^. At physiological conditions, duplex DNA is thermodynamically more stable than ssDNA. Therefore, in contrast to DNA unwinding, DNA rewinding is a thermodynamically downhill process and will, in principle, occur spontaneously. Nevertheless, DNA-rewinding enzymes are needed to either accelerate DNA duplex formation or catalyse a rewinding reaction coupled to another thermodynamically uphill reaction, such as DNA unwinding during branch migration and stalled replication fork rescue, or the displacement of bound proteins^9^.

In this work, we carried out a comparative study of two DNA helicases from the SF2 superfamily, the bacteriophage T4 UvsW and *Escherichia coli* RecG enzymes^3^,^4^. Enzymes from the SF2 superfamily have a crucial role in genome maintenance and, in humans, defects in different SF2 helicases, such as Bloom Syndrome helicase, Werner Syndrome helicase, Rothmund–Thomson syndrome or HARP (Schimke immuno-osseous dysplasia) can lead to different genetic disorders^10^. Both RecG and UvsW catalyse the 3′–5′ directional unwinding of DNA structures that mimic intermediates involved in DNA replication, recombination and repair^3^,^4^. RecG is necessary for efficient recombination-dependent DNA repair *in vivo*^11^. UvsW is a functional analogue of RecG, as it can complement some of the defects of a *recG* null^12^. In addition, both helicases have central roles in the rescue of stalled replication forks^13^,^14^, reforming template duplex DNA by rewinding ssDNA regions exposed at DNA forks^15^,^16^.

Here the DNA rewinding activities of RecG and UvsW are investigated using single-molecule force manipulation techniques to apply tension at the extremities of DNA hairpin substrates (Figs 1a and 2a)^17^. By applying a force of ∼15 pN, the hairpin is partially denatured. The rewinding reaction catalysed by either RecG or UvsW motors can then be followed by monitoring changes in the extension of the DNA molecule (*Z*_e_(t)). This approach permits a detailed real-time analysis of DNA rewinding—a downhill process that has proved difficult to characterize biochemically in ensemble assays. The same assay also allows simultaneous testing of the enzyme-unwinding activities. We find that despite their low sequence and structural homologies^18^,^19^, both enzymes present very similar activities. They exhibit a fast and processive DNA-rewinding activity against high denaturing forces (up to 35 pN), revealing an active rewinding mechanism. Moreover, both enzymes catalyse DNA rewinding when coupled to DNA unwinding during fork regression and branch migration. However, they do not catalyse efficient unwinding uncoupled from rewinding. Altogether, the results show that RecG and UvsW are robust DNA-rewinding enzymes, whose ability to generate work during the rewinding reaction leads to efficient DNA unwinding and protein-displacement activities. Despite their distinct preferences for branched DNA structures, both enzymes interact in similar manner with the fork, suggesting that the RecG-fork-regression mechanism proposed previously^18^, which utilizes DNA rewinding, also holds true for the UvsW motor.

## Results

### Single-molecule experimental configurations

**Figure 1:**
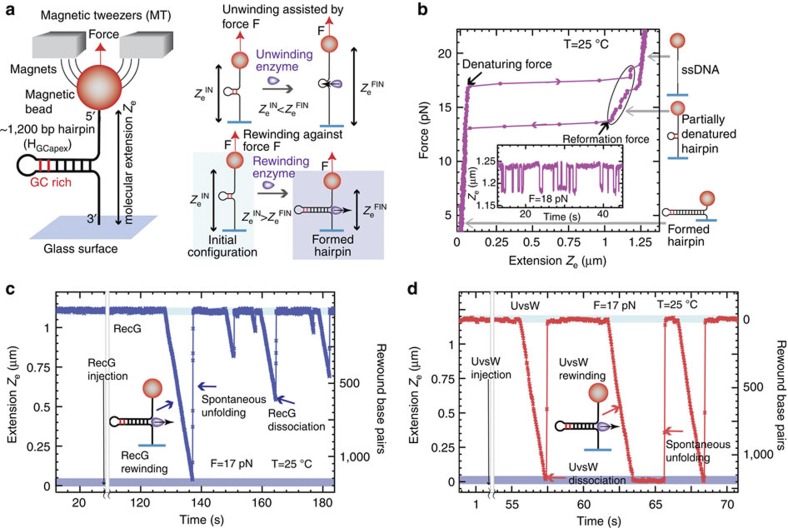
Efficient rewinding of DNA is monitored in real time using magnetic tweezers. (**a**) Schematic of the MT experimental setup and progress of the reaction. A DNA hairpin substrate was tethered between a glass surface and a magnetic bead held in a magnetic tweezers. The GC-rich region of the H_GCapex_ hairpin is shown in red. The DNA rewinding or unwinding reaction was followed by monitoring changes in the distance between the bead and the glass surface (*Z*_e_). (**b**) Hairpin configurations can be precisely followed using MT, and typical data in the absence of protein are shown. The force as a function of *Z*_e_ for the H_GCapex_ hairpin at 25 °C demonstrated stable hairpin folding below 15 pN and mechanical unfolding above 17 pN. Inset shows the *Z*_e_(t) at 18 pN where the molecule hops between the fully open (that is, completely denatured) and partially denatured hairpin configurations (**c**,**d**). Representative traces of rewinding reactions catalysed by RecG (**c**) and UvsW (**d**) enzymes. Reactions were carried out as described in Methods. Rewinding bursts were detected as decreases in *Z*_e_. The molecular extensions corresponding to the initial partially denatured hairpin and the final fully formed hairpin are highlighted in light and dark blue, respectively.

**Figure 2:**
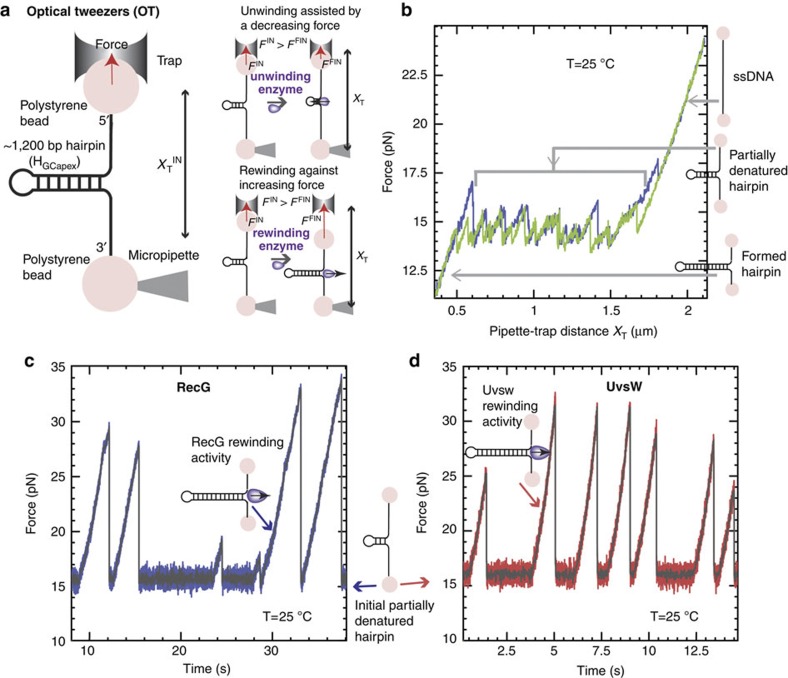
Efficient rewinding of DNA at very large forces is monitored in real time using optical tweezers. (**a**) Schematic of the OT experimental setup and progress of the reaction. A DNA hairpin substrate was tethered between two beads—one trapped in the optical trap and the other fixed in a tip of a micropipette. In this passive configuration, the DNA rewinding or unwinding reaction was followed by monitoring changes in force. (**b**) Hairpin states can be precisely followed using OT, and typical progress curves in the absence of protein are shown. The force as a function of *X*_T_ for the H_GCapex_ hairpin at 25 °C shows a broad region resulting in a partially unzipped hairpin configuration. (**c**,**d**) Representative traces of rewinding reactions catalysed by RecG (**c**) and UvsW (**d**) proteins. Experimental traces showing force as a function of time starting with the initial partially denatured hairpin configuration. Rewinding bursts were detected as force rips.

We used magnetic and optical traps (Figs 1a and 2a) to manipulate a 1,200-base pair (bp) DNA hairpin and investigated the rewinding, unwinding and branch migration activities of UvsW and RecG. Reaction progress was deduced from changes in the extension of the DNA molecule, *Z*_e_, in magnetic tweezer (MT) assays, or from changes in the force applied in optical tweezer (OT) assays. Experiments were carried out by tethering the DNA hairpin substrate between a glass surface and a magnetic bead (MT setup) or between two polystyrene beads (OT setup). In the MT setup (Fig. 1a), a controlled force was applied to the ends of the hairpin by using two magnets, and *Z*_e_(t) was measured by tracking the position of the magnetic bead^20^. In the OT setup (Fig. 2a), one bead was held in the optical trap, whereas the other was fixed on the tip of a micropipette. The applied force (related to the displacement of the trapped bead) was deduced from the measurement of the light deflected by the bead in the trap^21^. DNA rewinding (unwinding) could be detected as a decrease (increase) in *Z*_e_ or an increase (decrease) in the applied force in MT and OT setups, respectively.

### Rewinding DNA while working against an opposing force

To investigate unwinding and rewinding, we first pulled the extremities of the tethered DNA molecule to partially unzip the hairpin structure (Figs 1a and 2a). As in previous work^17^,^22^, we used a DNA hairpin containing a GC-rich region before the apex (H_GCapex_). By applying a force of 17 pN with MT we generated partially denatured hairpins with the initial stem unzipped but the GC-rich region intact (Fig. 1b). This partially denatured hairpin substrate allowed the testing of both unwinding and rewinding activities. Note that in this design, the applied force destabilized the DNA duplex and therefore assisted unwinding but hindered rewinding. Addition of RecG·ATP or UvsW·ATP only produced bursts of DNA rewinding, detected as a transient decrease in *Z*_e_ (Fig. 1c,d). After enzyme dissociation, the spontaneous force-induced DNA unzipping was observed as a sudden and rapid recovery of the initial molecular extension. The conversion of changes in molecular extension to number of bps rewound was performed by measuring the elastic response of ssDNA (Supplementary Fig. S1). The rewinding rates at denaturing force (∼17 pN) measured from these assays were 123±2 and 715±10 bp s^−1^ at 25 °C for RecG and UvsW, respectively, and nearly doubled when the temperature was raised to 37 °C (Supplementary Fig. S2). Both helicases presented a DNA-rewinding activity with a high processivity at the denaturing force of 480±20 bp for RecG and 9±1 Kbp for UvsW and, as expected, the rewinding reactions were ATP-dependent (Supplementary Fig. S2).

### Dissociation from the DNA at high forces without stalling

**Figure 3:**
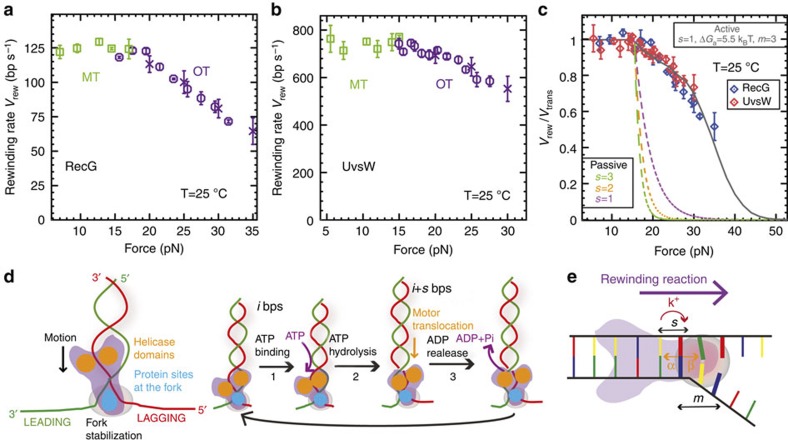
RecG and UvsW catalyse efficient rewinding reactions against large opposing forces. (**a**,**b**) Mean rate of rewinding as a function of force for RecG (a, number *n* of experimental traces analysed from 56 to 428 depending on the conditions) and UvsW (b, *n* from 37 to 214 depending on the conditions) at 25 °C. Reactions were carried out using MT (green squares) for forces ≤15 pN and OT (purple circles and crosses) for opposing forces ≥15 pN. Rates at high forces are computed as described in Methods both from OT-passive data (Fig. 2, purple circles) and from OT force-jump data (Supplementary Fig. S3, purple crosses). Error bars are s.e.m. (**c**) The experimentally measured rate of rewinding for RecG (blue circles) and UvsW (red diamonds) normalized to the maximum rate of rewinding as a function of the force. Error bars are s.e.m. The results are compared with the predictions obtained from the extended model for a passive enzyme with different step sizes and for an active enzyme model that agrees very well with the experimental results (*s*=1, *ΔG*_*a*_=5.5 *k*_B_*T*, *m*=3). (**d**) Rewinding scheme based on that previously proposed for RecG fork regression^18^. Helicase domains are marked in yellow, whereas the protein region that makes contact with the fork is shown in blue (for example, wedge domain of RecG). Along the ATP cycle at least three different states can be considered: protein without nucleotide bound (1), protein with ATP bound (2) and protein with ADP bound (3). (**e**) Schematics of the extended model used to describe the UvsW and RecG rewinding behaviours: α and β are the base pair opening and closing rates; *k*^*+*^ is the forward translocation rate; *s* is the enzyme step size and *m* is the range of the protein–DNA interaction potential (in the figure *s*=1 and *m*=3).

The force generated by these molecular motors during the DNA-rewinding process (or robustness) was investigated by examining the rewinding activity of UvsW and RecG over a wide range of applied forces using OT. In OT assays, we initially increased the distance between the micropipette and the trap (*X*_T_) until reaching a force of ∼16 pN and partially unzipping the hairpin (Fig. 2b). With the addition of UvsW·ATP or RecG·ATP at a constant *X*_T_, the rewinding reaction caused shortening of the molecule that induced the displacement of the bead in the trap, generating an increase in tension. Consequently, in this OT-passive configuration, the opposing applied force increased as DNA rewinding proceeded, allowing for testing the stalling behaviour of the motor^23^,^24^. The bursts of rewinding were then detected as force rips, in which the opposing force increased from 16 to 20–35 pN (Fig. 2c,d). After the dissociation of the enzyme from the DNA junction, the initial hairpin configuration was restored and the force dropped instantaneously to the initial value of 16 pN. By using the previously measured elasticity of ssDNA, we computed the rewinding rate as a function of the opposing force (see Methods). Both the RecG- and UvsW-catalysed DNA-rewinding reactions proceeded against forces of 30–35 pN, with only a moderate drop (about 40%) in the reaction rate (Fig. 3a,b) suggestive of powerful molecular motors. However, the enzyme processivities were strongly dependent on the force (Supplementary Fig. S3). Above 35 pN, the molecule rapidly unzipped, revealing that at such high-force regime both enzymes dissociated from the DNA junction without stalling.

### DNA unwinding is seen only when coupled to rewinding

Even though partially denatured hairpins are substrates for testing both rewinding and unwinding activities (Figs 1a and 2a), RecG and UvsW only catalysed DNA hairpin rewinding but not unwinding (Figs. 1c,d and 2c,d), demonstrating the preference for these enzymes for rewinding over unwinding in an agreement with the limited unwinding observed in previous works^3^,^17^,^25^. However, both RecG and UvsW were able to couple rewinding to unwinding in regressing forks. DNA forks were generated and maintained at a moderate force (between 5 and 13 pN), with MT using hybridization of short oligonucleotides to transiently block the fork (see below and Supplementary Fig. S4). Addition of UvsW or RecG and ATP promoted fork regression via unwinding of the oligonucleotide concomitant with rewinding of the hairpin, although hairpin unwinding was never observed (Supplementary Fig. S4d), supporting the idea that these enzymes only perform efficient unwinding when coupled to rewinding. After the displacement of the bound oligonucleotide, the rewinding of the hairpin proceeded at a constant rate. Note that, below the refolding force (∼15 pN), the rewinding reaction is thermodynamically favoured. In these conditions, rewinding is limited by the enzyme translocation, which necessarily implies an interaction between the helicase and at least one of the fork tails, inhibiting the instantaneous reformation of the hairpin expected below the refolding force. This rewinding rate at low forces then corresponds to the maximum translocation rate of the enzyme (Fig. 3a,b). Slow DNA rewinding limited by enzyme motion had been observed in single-molecule studies of the ssDNA translocation activity of several helicases^26^,^27^,^28^.

### Active enzymes that generate work during DNA rewinding

Mechanical manipulation is a powerful tool to investigate the mechanisms of molecular motors^29^,^30^. In particular, the study of DNA unwinding by helicases assisted by mechanical forces has allowed the discrimination between passive and active mechanisms^26^,^27^,^31^,^32^. A similar analysis can be carried out for rewinding enzymes by measuring the reaction rate against the applied opposing forces. Remarkably, when normalized to the translocation rate (measured as the rewinding rate at low forces), both enzymes presented the same dependence on the applied force (Fig. 3c), supporting the idea that RecG and UvsW must employ analogous rewinding mechanisms. We extended the model proposed by Betterton *et al*.^33^,^34^ for helicases to describe DNA-rewinding motors in presence of mechanical opposing forces using the DNA substrate-bound RecG crystal structure as a reference^18^. Recent bulk studies on RecG^35^,^36^ and our results below also support such DNA/protein organization, in which the helicase-motor domains orient towards the duplex DNA and a so-called wedge domain interacts with the junction (Fig. 3d). The proposed model is depicted in Fig. 3e. The motor moves along the duplex in the forward direction (towards the junction) at a constant rate *k*^*+*^, whereas the dynamics of the junction is governed by the base-pair opening and closing rates α and β. These rates verify α/β=exp(−Δ*G*_bp_(*f* )), where Δ*G*_bp_(*f* ) represents the free energy difference between the base pair opened and closed conformations at a given opposing force *f*. The motor, which moves in steps of *s* nucleotides, promotes rewinding by stabilizing the *m* base pairs close to the fork by an energy Δ*G*_*a*_ (see Methods for the details of the model). Briefly, at low opposing forces the motor can move at the maximum speed *k*^+^ because DNA rewinding is a thermodynamically downhill process (that is, β>>α). However, when the opposing force increases above the denaturing force, the DNA fork presents a barrier to the movement of the motor (β<α). Consequently, the rewinding rate decreases below *k*^+^. The extent of the slowing depends on the ability of the enzyme to stabilize the DNA duplex. Active and passive enzymes are characterized, respectively, by large and small values of Δ*G*_*a*_ as compared with the free energy of formation of a base pair^31^ (∼2.5 *k*_B_*T* under the experimental conditions, see Methods). A passive rewinding enzyme (Δ*G*_*a*_=0) would only rely on trapping the spontaneous and transient formation of the base pairs at the fork, whereas an active rewinding enzyme would directly stabilize the DNA fork (Δ*G*_*a*_>0), promoting duplex formation in a more efficient way. Our experimental results are inconsistent with a passive scenario and can only be reproduced by an active model with similar mechanistic characteristics for both enzymes: a step size of one or two base pairs and a stabilization base pair energy of about 5 *k*_B_*T* (Fig. 3e and Supplementary Fig. S5), revealing their strong active character. The active and passive models tested here are analogous to the active disruption model and the Brownian ratchet model proposed for hexameric branch migration helicases^37^.

The maximum work performed by the enzyme to rewind a single base pair is given by: 
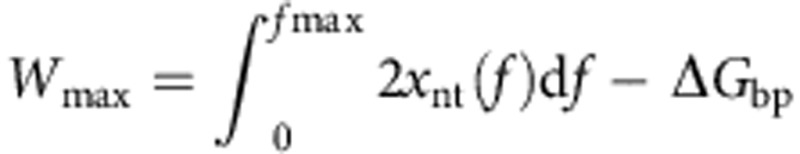
, where the first term accounts for the stretching energy required to bring together the two nucleotides at the junction (with *x*_*nt*_ (*f* ) corresponding to the extension of a single nucleotide at a given force *f* ) and the second term, Δ*G*_bp_, is the free energy of formation of a base pair. The former can be directly measured from the ssDNA elasticity and the latter can be estimated from thermal or force denaturation data (Supplementary Fig. S1). Taking *f*_max_ ∼35 pN, *W*_max_ results in 7.5 *k*_B_*T*, a value close to the energy associated with the hydrolysis of an ATP molecule (Δ*G*_ATP_∼20 *k*_B_*T*), which gives an upper limit for the motor step size *s* ≤
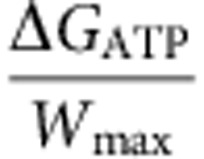
∼2 bp in agreement with the estimation obtained from the modelling (*s*=1–2 bp), but smaller than the 4-bp step size proposed for *Thermotoga maritime* RecG^36^. This analysis leads to an estimation for the motor efficiency 
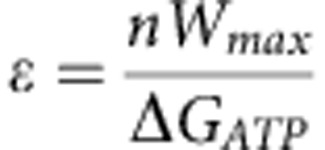
 between 40 and 75%.

### Regression of stalled forks with a nascent strand

**Figure 4:**
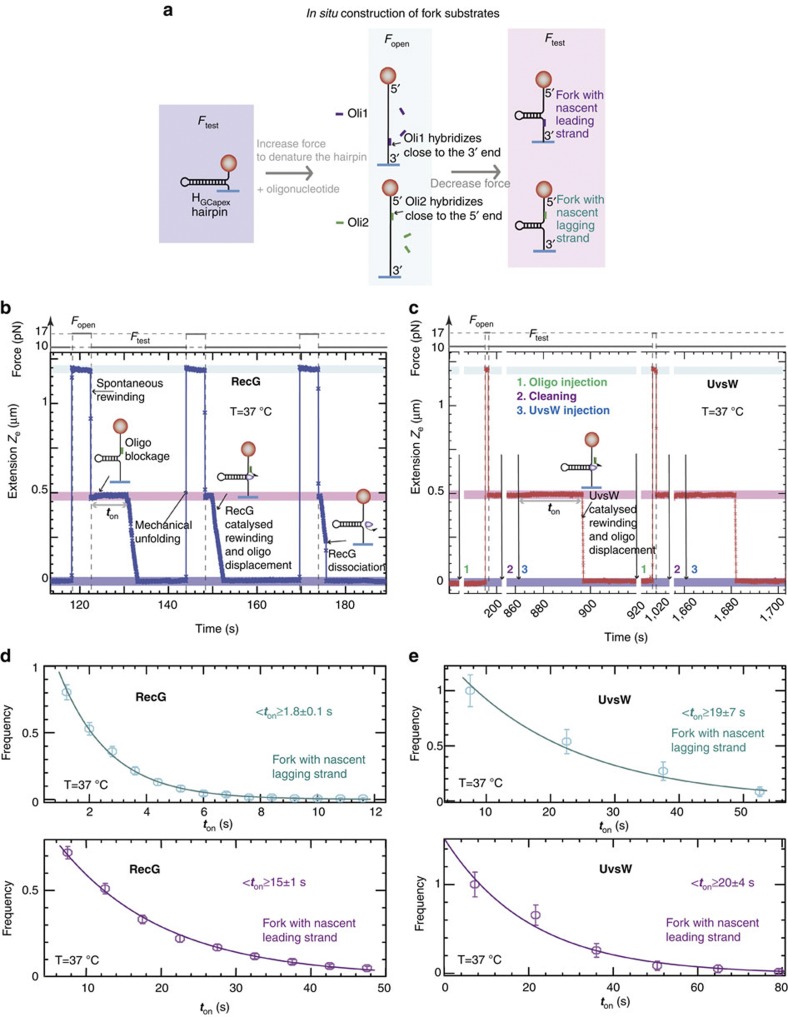
Substrate requirements for RecG- and UvsW-catalysed fork regression are distinct. (**a**) Schematics of the forked-DNA substrate construction with nascent lagging or leading strand from the H_GCapex_ hairpin. (**b**,**c**) Experimental traces showing the cyclic fork regression assay for RecG (**b**) and UvsW (**c**) proteins at 37 °C. The molecular extensions corresponding to the initially formed hairpin, the totally denatured substrate and the partially denatured hairpin are highlighted in dark blue, light blue and pink, respectively. For RecG, a single injection of oligonucleotide and enzyme is sufficient, whereas for UvsW measurements three separate injections are needed: first oligonucleotide injection, second buffer injection and third UvsW injection. (**d**,**e**) The distributions of enzyme-binding times for RecG (**d**), (number *n* of experimental traces analysed from 427 to 566 depending on the conditions) and UvsW (**e**), (*n* from 55 to 86 depending on the conditions) and different fork geometries. Error bars are inversely proportional to the square root of the number of points for each bin.

Next, we investigated the ability of RecG and UvsW to regress forks with either lagging or leading nascent strands using MT. In these assays, DNA forks were generated with a 90-mer oligonucleotide, which mimics the nascent strand, hybridized to either the leading or the lagging strand and using force modulation (Fig. 4a). At applied forces >2 pN and in the absence of protein, the hybridized oligonucleotides produced a permanent block and the fork structure was maintained. Addition of UvsW or RecG and ATP lead to the recovery of the formed hairpin in two phases: first, fork regression via simultaneous rewinding of the hairpin and displacing of the hybridized oligonucleotide, second, final hairpin rewinding after oligonucleotide departure. By repeating the force modulation in the presence of oligonucleotides and enzyme, oligonucleotide hybridization and fork regression could be cycled in a single experiment (Fig. 4b,c). Even though both enzymes translocate on ssDNA^38^,^39^, we found that excess oligonucleotide inhibited fork binding of UvsW only. Consequently, the cyclic fork-regression assays for UvsW required sequential injections of oligonucleotide and enzyme separately, whereas RecG and oligonucleotide could be added simultaneously. These results demonstrate that RecG binds to model fork structures with very high affinity relative to ssDNA^40^.

Previous studies on RecG, employing forks with heterologous arms to prevent spontaneous branch migration, demonstrated a preferential unwinding of forks with lagging nascent strand^4^,^40^. Here we investigated the preference of these enzymes for a special fork geometry using homologous junctions whose structures could be maintained by the applied force. Under these conditions, we compared the time required for the initiation of the fork regression that reflects the protein-binding time *t*_on_ with different fork geometries, measured from the cyclic fork-regression assay. The analysis shows that RecG binds ten-fold faster to forks with lagging nascent strand than to forks with leading nascent strand (Fig. 4d). In contrast, UvsW bound with a similar rate to either fork substrate (Fig. 4e). These measurements allow the estimation of *k*_on_ for binding forks with nascent lagging (*k*_on1_) or leading (*k*_on2_) strand, where *k*_on_=〈*t*_on_〉^−1^[E]^−1^and [E] is the enzyme concentration, leading to *k*_on1_∼ 6 × 10^7^ M^−1 ^s^−1^ and k_on2_∼7 × 10^6^ M^−1 ^s^−1^ for RecG and *k*_on1_=*k*_on2_∼5 × 10^6^ M^−1 ^s^−1^ for UvsW.

### Mapping the interaction of the enzymes with the DNA fork

A set of experiments was designed to map the interactions of both RecG and UvsW with DNA using modified DNA forks (Supplementary Fig. S6)^4^. We prepared forks presenting a polarity block (that is, a region with the polarity of the phosphate backbone reversed) in either the leading or the lagging strand and located at different positions with respect to the ssDNA/dsDNA junction (Supplementary Fig. S7a). Experiments with such modified forks showed that RecG and UvsW interacted with both ssDNA tails at the junction and with the parental duplex DNA along the first 20 bp (RecG, Supplementary Fig. S7b) or 10 bp (UvsW, Supplementary Fig. S7c), in agreement with DNA footprinting results^41^. Overall, these results support a mechanism consistent with the structural data for RecG^18^ in which the helicase-motor domains track along the lagging strand on the parental duplex region towards the junction (3′–5′), whereas other parts of the protein maintain contacts with the template arms at the junction (Fig. 3d).

### Fork regression and fork reversal activities depend on Mg^2+^

**Figure 5:**
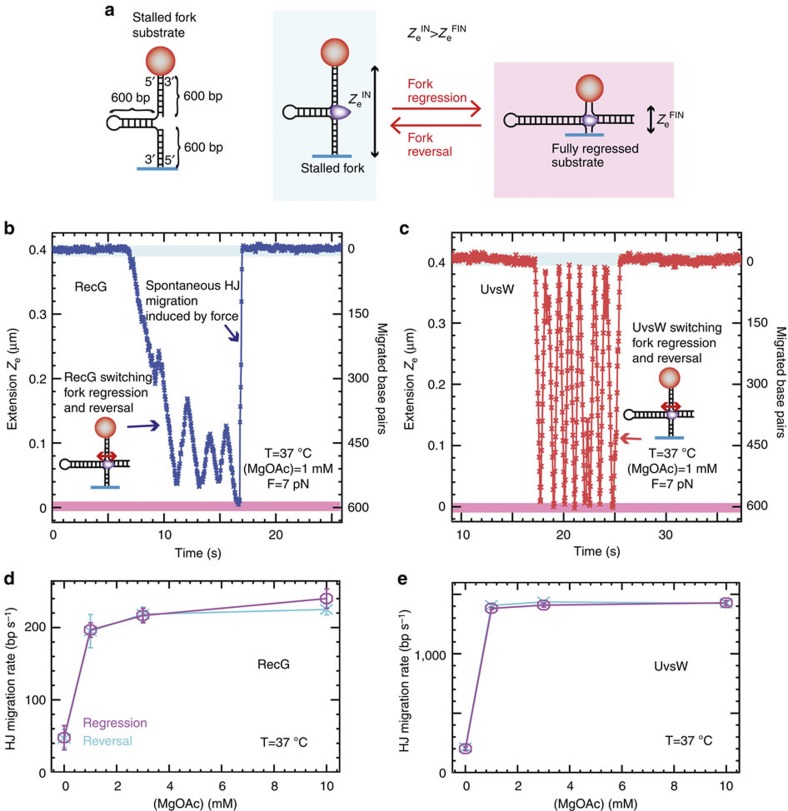
RecG and UvsW catalyse the Holliday junction formation and migration from a stalled fork intermediate. (**a**) Schematic of the stalled fork substrate and the fork regression and HJ branch migration reaction. (**b**,**c**) RecG and UvsW branch migration traces performed in a buffer containing 1 mM MgOAc at 37 °C. The molecular extensions corresponding to the initial stalled fork and the final fully regressed configurations are highlighted in light blue and pink, respectively. (**d**,**e**) RecG (**d**, number of experimental traces analysed *n* from 64 to 126 depending on the conditions) and UvsW (**e**, *n* from 53 to 209 depending on the conditions) mean branch-migration rate during fork regression (magenta) and fork reversal (cyan) at 7 pN as a function of the MgOAc concentration. Error bars are s.e.m.

**Figure 6:**
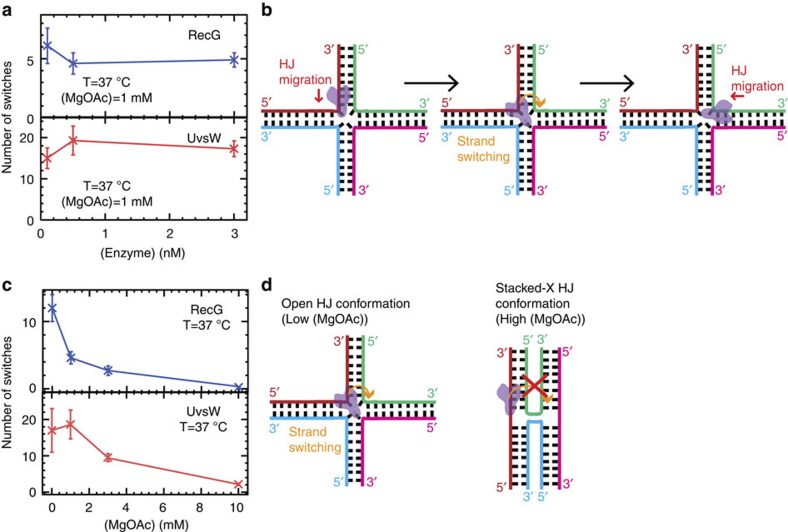
Switches in the direction of HJ branch migration catalysed by single RecG and UvsW enzymes are dependent on the ionic strength. (**a**) The mean number of switches in the HJ branch-migration direction catalysed by RecG (number *n* of experimental traces analysed from 25 to 76 depending on the conditions) and UvsW (*n* from 17 to 68 depending on the conditions) at 37 °C, 1 mM MgAc and 7 pN as a function of the enzyme concentration. Error bars are s.e.m. The average number of switches remains constant when changing the enzyme concentration (from 0.1 to 3 nM), showing that the HJ branch-migration switching is mediated by a single enzyme (RecG or UvsW). (**b**) Schematics of the enzyme strand-switching mechanism, which can lead to changes in direction of the HJ branch migration. (**c**) The mean number of switches in the HJ branch-migration direction catalysed by RecG (*n* from 33 to 74 depending on the conditions) and UvsW (*n* from 17 to 41 depending on the conditions) at 7 pN and 37 °C as a function of the MgOAc concentration. Error bars are s.e.m. (**d**) Schematics of the open and X-stacked HJ conformations favoured at low and high ionic strength, which, respectively, facilitate and hinder the enzyme strand-switching transition.

We recently developed an assay to generate a DNA substrate mimicking a stalled fork *in situ*, which allowed us to monitor UvsW-dependent Holliday junction (HJ) formation and its subsequent branch migration in real time^17^. This study revealed that UvsW frequently switches the direction of branch migration between fork regression and fork reversal—a property that turns out to be crucial for remodelling stalled replication forks leading to the resumption of DNA replication^17^,^42^. Here we performed HJ generation and migration assays with a stalled fork substrate (with 600-nt long nascent strands, Fig. 5a) using UvsW and separately, RecG. The regression of the preformed fork was initiated by the addition of enzyme·ATP. Fork regression and reversal were followed by monitoring *Z*_e_(t) (Fig. 5b,c). The initial transient decrease in *Z*_e_ corresponded to the formation and migration of the HJ, until the fork had been partially or totally regressed. The subsequent increase in *Z*_e_ corresponded to the migration of the HJ towards fork reversal. Note that, in our experimental configuration, the application of the force at the extremities of the fork favoured reversal over regression. Therefore, spontaneous fork reversal occurred whenever the enzyme dissociated from the DNA (Fig. 5b and Supplementary Fig. S8). The rates of RecG- and UvsW-catalysed branch migration during fork regression and fork reversal were mostly independent of the applied force and close to the enzyme-catalysed rates of DNA rewinding (Supplementary Fig. S8) but were dependent on the Mg^2+^ ion concentration (Fig. 5d). The switches in the HJ branch-migration direction were independent of the enzyme concentration (Fig. 6a), strongly suggesting that they were mediated by a single enzyme complex probably via a strand-switching mechanism (Fig. 6b). Interestingly, the ability of both enzymes to switch the direction of the HJ branch migration was strongly dependent on the Mg^2+^ ion concentration (Fig. 6c). This phenomenon might be related to changes in the HJ conformation controlled by the divalent metal ion concentration^43^,^44^; the open and stacked-X HJ conformations favoured at low and high divalent ion concentrations, respectively, might facilitate and hinder the enzyme strand switching at the junction (Fig. 6d).

## Discussion

The primary conclusions of this study are that both RecG and UvsW catalyse robust DNA-rewinding reactions that are critical to stalled DNA replication-fork rescue^42^,^45^. DNA rewinding, a thermodynamically downhill process, is coupled to either DNA unwinding (Fig. 5) or protein displacement (Supplementary Fig. S9)^17^,^46^—two endergonic reactions. Despite their differences in structure and sequence homologies^18^,^19^, UvsW and RecG present similar behaviour with processive and fast DNA-rewinding activities against large applied forces of up to 35 pN, with only a moderate reduction in the rate of rewinding. These results demonstrate that both enzymes use an active mechanism of rewinding in which the interaction of the enzyme with the DNA at the fork stabilizes the base pairs at the junction, promoting rewinding and generating work of about 8 *k*_B_*T* per rewound base pair (Fig. 3). Our assays with a stalled fork substrate or substrates coated with ssDNA-binding protein (Fig. 5 and Supplementary Fig. S9)^17^,^46^ prove that RecG and UvsW can efficiently use this work to unwind nascent strands or to displace bound proteins—processes requiring an energy input of few *k*_B_*T* (1–3 *k*_B_*T*) for unwinding a single bp or several *k*_B_*T* for displacing DNA-binding proteins^21^,^47^,^48^. The active mechanism for fork regression is in stark contrast to the passive or Brownian ratchet model proposed for hexameric branch-migration helicases^37^, such as RuvAB, or DnaB^49^,^50^, in which the enzyme uses unidirectional motion to trap spontaneous bp fraying. This difference might arise from the role of the protein domain located at the DNA junction in RecG-like helicases (so-called wedge domain)^18^, which is not present in the case of hexameric helicases^51^. Therefore, we conclude that the mechanisms used for monomeric RecG-like helicases and hexameric helicases during rewinding and fork regression are different.

Previous work proposed a DNA-rewinding model for UvsW involving a fixed contact between the protein and one of the DNA strands while translocation occurred along the opposite DNA strand^3^. Our results showing that UvsW rewinds hairpins against denaturing forces (Figs 1 and 2) imply simultaneous translocation of the enzyme on both strands of DNA and are inconsistent with such a model. Further, by applying forces ≥18 pN and by completely disrupting the hairpin structure including the GC-rich apex region, rewinding was totally inhibited, suggesting that the enzyme requires binding to a duplex region of the substrate to initiate the reaction. This is consistent with SF2 helicases maintaining their interactions with DNA substrates through the phosphate backbone of DNA and hence capable of translocation on both ssDNA and dsDNA structures tracking on the phosphate backbone^52^. Indeed, experiments performed with DNA substrates with regions of reverse backbone polarity (Supplementary Fig. S7) demonstrated the importance of interactions between UvsW, the parental duplex DNA and both fork tails. Critically, these protein–DNA interactions are analogous to those of RecG^4^,^18^, suggesting that UvsW assembles on DNA forks in a manner similar to RecG. Moreover, we found that the interactions of these proteins with the parental duplex DNA are more pronounced with the lagging strand tail, supporting a rewinding mechanism based on the fork-regression model proposed by Singleton *et al*.^18^ (Fig. 3d): the core helicase domains (shown in yellow) interact with the parental duplex DNA via the phosphodiester backbone and move along the lagging strand towards the dsDNA/ssDNA junction with a 3′ to 5′ polarity, whereas other regions of the protein (shown in blue) interact with both DNA template arms at the junction promoting rewinding. The fact that such a scheme applies to two enzymes with low structural homology suggests that this might be a general mechanism for monomeric SF2 family-rewinding motors.

One of the main roles associated with both RecG and UvsW enzymes is the rescue of stalled DNA replication forks via fork regression^13^,^14^,^15^,^17^. Here we demonstrate that both enzymes catalyse a set of reactions relevant to stalled DNA-replication fork rescue. First, both helicases efficiently catalyse coupled DNA unwinding and rewinding. These activities are used to regress a fork away from the site of DNA damage. Second, once the forks have been regressed into a HJ structure, each helicase is capable of driving branch migration. Third, the enzymes display the ability to switch the direction of HJ branch migration and to restore the fork to its initial configuration (Figs 5 and 6). Our enzyme-concentration analysis indicates that the RecG- and UvsW-catalysed switches in HJ branch-migration direction are mediated by a single-enzyme complex, probably via a strand-switching mechanism^28^. High concentration of divalent ions, which is known to induce the transition from the open to the X-stacked HJ conformation^43^,^44^, inhibits the switching behaviour for both RecG and UvsW, showing that this reaction is very sensitive to the DNA structure at the junction. Our recent findings showed that UvsW in collaboration with the T4 holoenzyme was able to overcome a DNA lesion by regressing the stalled forks and bypassing the lesion via a template-switching pathway^17^. The observed analogous activities of both these enzymes strongly suggest that RecG could also have the same role in *E. coli*. We also found that RecG binds preferentially to forks with a lagging nascent strand (Fig. 4). This binding specificity might allow RecG to efficiently target asymmetric forks for remodelling by promoting either lesion bypass or lesion excision repair^42^,^45^. In contrast, UvsW binds with similar affinity to different DNA substrates, which might confer a wider functional diversity necessitated by the rapid phage life cycle.

## Methods

### Proteins and buffer

The T4 UvsW helicase was purified as follows. The pET28 vector carrying the gene for UvsW with a *N*-terminal His_6_-tag was transformed into BL21(DE3) and selected on agar plates containing 50 μg ml^−1^ kanamycin. A single colony was selected from the agar plate and grown overnight at 37 °C in LB media containing kanamycin (50 μg ml^−1^). A 10 ml of the overnight inoculum was diluted into 1 l of LB with kanamycin. The cells were grown at 37 °C until the OD_600 nm_ reached ∼0.5. The temperature of the culture was dropped to 18 °C and the protein expression was induced by the addition of 0.2 mM isopropyl-1-thio-β-d-galactopyranoside. The cells were then grown for an additional 10–16 h at 18 °C after which the cells were pelleted by centrifugation in a Beckman centrifugation device at 4225 × *g* at 4 °C for 15 min. The cells were resuspended in 10 ml of 20 mM Tris–HCl, 500 mM NaCl and 5 mM imidazole (pH 8.0) buffer for every gram of the cell pellet in the presence of EDTA-free Complete protease inhibitor cocktail from Roche Diagnostics. The resuspended cells were then lysed using sonication and centrifuged at 15,000 r.p.m. for 45 min at 4 °C. The lysate was then loaded on a nickel nitrilotriacetic acid (Ni-NTA)-agarose column, washed with 10 column volumes of lysis buffer containing 20 mM imidazole, 1 M NaCl and the protein eluted with lysis buffer containing 100 mM imidazole. The eluted protein was diluted three-fold with a buffer containing 20 mM Tris–HCl (pH 7.5), 400 mM NaCl and loaded on a 20-ml P11 phosphocelluose column. The column was washed with 10 column volumes of the same buffer before eluting UvsW with a linear gradient of 0.4–1.2 M NaCl. The fractions were analysed on 12% SDS–PAGE and the fractions containing UvsW were pooled together and concentrated to 22 μM using an Amicon centrifugation device with a 30 kD cutoff and flash-frozen in liquid N_2_ in 10 μl aliquots at −80 °C. Protein concentration was calculated based on an extinction coefficient of 73,920 M^−1 ^cm^−1^.

The RecG protein was purified as described previously^39^,^40^. The first column was a 30-ml Q-Sepharose column equilibrated in Buffer A (20 mM Tris-HCl (pH 8.5), 1 mM EDTA, 1 mM dithiothreitol and 10 mM NaCl). The protein was eluted using a linear gradient (10–1,000 mM NaCl) with RecG eluting between 250 and 360 mM NaCl. The pooled fractions were subjected to heparin FF and hydroxylapatite chromatography as described^40^. Pooled fractions from the hydroxylapatite column were dialysed overnight into S Buffer (10 mM KPO_4_ (pH 6.8), 1 mM dithiothreitol, 1 mM EDTA and 100 mM KCl). The protein was applied to a 1-ml MonoS column and eluted using a linear KCl gradient (100–700 mM) with RecG eluting at 350 mM KCl. The fractions containing RecG were pooled and dialysed overnight against storage buffer (20 mM Tris-HCl (pH 7.5), 1 mM EDTA, 1 mM dithiothreitol, 100 mM NaCl and 50% (v/v) glycerol). The protein concentration was determined spectrophotometrically using an extinction coefficient of 49,500 M^−1 ^cm^−1^. The modifications to the purification procedure yielded a four-fold increase in specific activity relative to that used previously^40^.

Experiments were performed in buffer containing 25 mM TrisOAc (pH 7.50), 150 mM KOAc, 10 mM MgOAc, 1 mM dithiothreitol and 1 mM ATP. In the experiments where the effect of ATP (Supplementary Fig. S2) or divalent ions was examined (Figs 5 and 6 and Supplementary Fig. S8), ATP was varied from 0.01–1 mM, and MgOAc was varied from 0–10 mM. Experiments shown in Fig. 1 were performed at 25 °C in order to combine the results with those from the OT assays (Fig. 2) that were performed at ambient temperature. All the other MT assays were performed at 37 °C (Figs 4, 5, 6). Protein concentration in single-molecule assays varied from 0.3 to10 nM UvsW or RecG depending on the assay.

### DNA substrates

The 1,200-bp hairpin was constructed by ligating a 1.1-kbp segment to a DNA hairpin loop and a fork with the 5′ biotin label (Supplementary Fig. S6). The gp43(exo-) polymerase was used to fill in the overhang on the 3′ end of the fork and to incorporate multiple digoxigenin-labelled dUTP nucleotides. Details of the hairpin preparartion are given in Manosas *et al*.^53^ The three 250-bp short DNA hairpin substrates containing regions of reverse polarity in the DNA backbone were constructed as follows. Briefly, a fork structure formed by two partially annealed oligonucleotides (A-1 was 5′-biotinylated to allow for attachment to the magnetic bead and A-3) and a short-hairpin oligonucleotide (C) was annealed and ligated to the compatible ends of a 200-bp DNA fragment formed by annealed oligonucleotides (B-1 and B-2) (Supplementary Fig. S6); oligonucleotide sequences are given in Supplementary Table S1. The digoxigenin label was incorporated by annealing a primer (oligo A-2) to the template strand and filling in the overhang with Klenow Fragment (3′→5′ exo-) (New England Biolabs) in the presence of dUTP-digoxigenin (Roche). The hairpin products were purified with NucleoSpin Extract II Kits (Clontech).

Fork substrates for single-molecule branch migration assays were generated *in situ* in the reaction chamber (Supplementary Fig. S8)^17^ using the T4 holoenzyme^54^.

### MT assays

We used a PicoTwist MT instrument ( www.picotwist.com) to manipulate individual DNA hairpin molecules tethered between a glass surface at the 3′ end and a magnetic bead at the 5′ end. The glass surface was treated with anti-digoxigenin antibody (Roche) and passivated with bovine serum albumin (0.2%, Sigma Aldrich). Streptavidin-coated Dynal magnetic beads (Invitrogen) were 1 μm in diameter. The DNA hairpins were manipulated by capturing the bead in a magnetic trap generated by a pair of permanent magnets. The applied force was controlled by varying the distance from the magnets to the sample. Video microscopy was used to track the position of the magnetic bead in three-dimensions with nanometer resolution at 30 Hz, from which the extension of the DNA molecule and the strength of the stretching force were deduced^20^,^55^.

### OT assays

We performed measurements with a highly stable miniaturized optical dual-beam laser tweezer^21^. One bead was immobilized on the tip of a micropipette; the other bead was captured in an optical trap generated by two counter-propagating laser beams. The force acting on the bead could be directly measured from the change in light momentum deflected by the bead^56^. A steerable optical trap can be moved up and down along the vertical axis, changing the total distance between the micropipette and the trap *X*_T_.

### Single-molecule data analyses

MT (Fig. 1) and OT force-jump (Supplementary Fig. S3) raw data, corresponding to the real-time evolution of the DNA extension in nm, were converted into the number of base pairs rewound or base pairs migrated by UvsW or RecG using a calibration factor determined from the elastic properties of ssDNA and dsDNA (Supplementary Fig. S1). Instantaneous enzymatic rates were obtained from a linear fit to the traces filtered with a third-order Savitzky–Golay filter over a time window of 0.1 s. The histogram of the instantaneous rates was fit to Gaussian functions.

In the passive configuration (Fig. 2), OT raw data, corresponding to the real-time evolution of the force in pN, were converted into the number of base pairs rewound by solving:


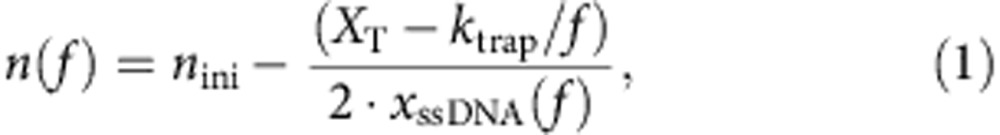


with *k*_trap_ being the stiffness of the optical trap, x_ssDNA_(*f* ) the extension of ssDNA per nucleotide at the force *f*, *X*_T_ the distance between the trap and the micropipette and *n*_ini_ the initial number of unzipped base pairs. As we initially trap the molecule in the configuration where the hairpin is unzipped except the last stretch rich of GC (40 bps), *n*_ini_ is estimated to be 1,200 bp. The value of the total distance *X*_T_, which is not directly measured, is computed from the initial value of the force *f*_ini_ and *n*_ini_ as:


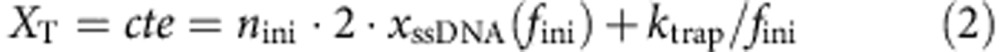


The converted traces *n(t)* were filtered with a third-order Savitzky–Golay filter over a time window of 0.03 s. Instantaneous enzymatic rates at a given force *f*_s_ were obtained as the slope of *n(t)* along the interval of points in the range {*f*_s_−0.25 pN <*f(t)* < *f*_s_+0.25 pN}. The histogram of the instantaneous rates was fit to Gaussian functions.

### Rewinding motor model

Betterton *et al*.^33^,^34^ proposed a framework for describing DNA unwinding by helicases. Here we extended this framework to DNA rewinding by rewinding motors using components of the model proposed for RecG based on structural data^18^. In the crystal structure of RecG complexed with a DNA fork, the helicase translocation domains are oriented towards the duplex, whereas a wedge domain interacts with the ssDNA/dsDNA junction, suggesting that enzyme translocation occurs along the duplex DNA, but interactions promoting rewinding might occur at the fork. This scheme is also in agreement with our results obtained with the reverse polarity substrates (Supplementary Fig. S7b,c), which identify protein interactions with both the DNA parental duplex and the ssDNA tails. Overall, the two scenarios, unwinding (or helicase activity) versus rewinding, mainly differ in three points: (1) the interaction between the enzyme and the duplex: an helicase destabilizes the duplex, whereas a rewinding motor stabilizes the duplex; (2) the preferential translocation direction: an helicase moves towards unwinding the fork, whereas a rewinding motor moves towards regressing the fork; (3) the substrate for translocation: the rewinding motor translocates along the parental duplex DNA, whereas helicase translocation occurs generally along one of the ssDNA tails. The model is depicted in Supplementary Fig. S5a. The motor moves forward along the duplex (towards the junction) or backwards at constant rates *k*^*+*^ and *k*^−^, whereas the dynamics of the junction is governed by the bp opening and closing rates α and β. As a first approximation, we have considered a fully unidirectional motor with *k*^−^=0. The motor, which moves in steps of *s* base pairs, stabilizes the duplex close to the protein/DNA duplex interaction site by an amount Δ*G*_*a*_ and within a range of *m* base pairs. The dynamics of the motor and the junction are linked in the following manner: the motor can only move forward (advance towards the junction) when the next *s* base pairs are formed and the junction can move backwards (opening of one base pair) when the enzyme is at least one nucleotide away from the junction.

The dynamics of the rewinding motor at a DNA fork is governed by the master equation for the probability *p*_*j*_ that the enzyme is *j* bases away from the DNA fork (*j* >0):


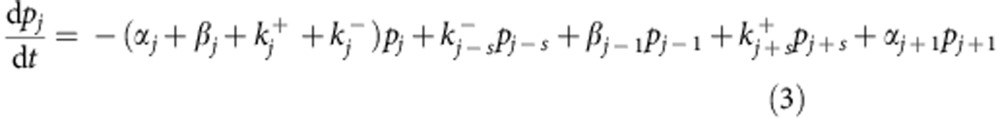


The bp opening and closing rates *α* and *β* read as:


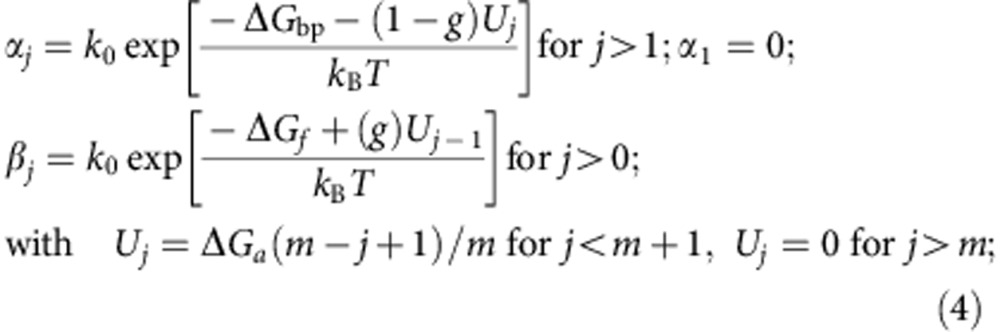


where *k*_0_ is the attempt frequency, Δ*G*_bp_ the base pair-free energy, Δ*G*_f_ the reduction in the free energy due to the external force (estimated from the ssDNA elasticity measured experimentally as 
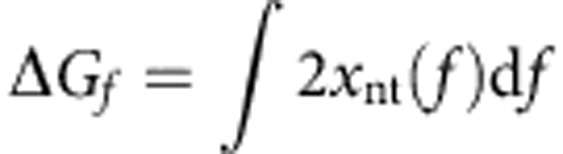
, where *x*_nt_ corresponds to the extension of a single nucleotide at a given force *f*, Supplementary Fig. S1) and *g* defines how the interaction potential affects the bp opening and closing kinetics^33^. The choice of such rates (equation (4)) is based on the description of the folding/unfolding kinetics of DNA molecules under the force given elsewhere^57^. Note that we have chosen a staircase potential of range *m* and amplitude Δ*G*_*a*_ (Supplementary Fig. S5b). The particular case where Δ*G*_*a*_=0 corresponds to the description for a passive rewinding motor. Following the description by Betterton and Jülicher, the forward *k*^+^ and backward *k*^−^ rates can be written as:


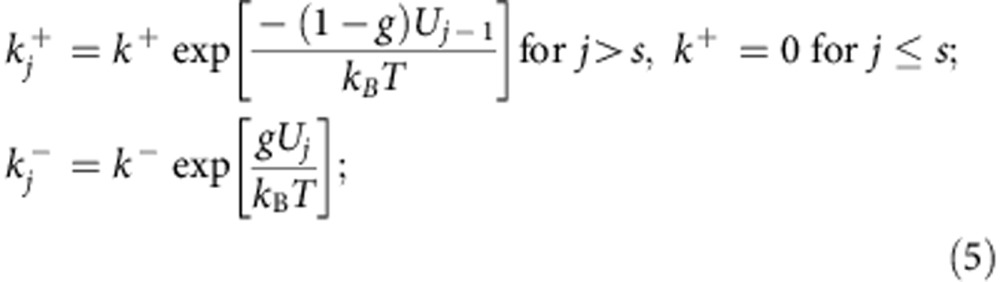


We performed Monte Carlo simulations of the model using the rates given in equations (4) and (5). We estimated Δ*G*_bp_ to be 2.5 *k*_B_*T* from the Boltzmann average of the base pair free energies (Δ*G*_*k*_) of the sequence under study (*k*) at the experimental temperature and salt conditions using the DNAMelt web server^47^, Δ*G*_bp_=*k*_B_Tln(
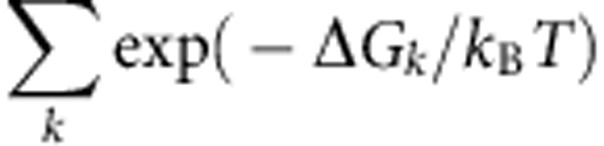
). In all simulations, we have fixed the attempt frequency to *k*_0_=10^6^ s^−1^, value consistent with the nuclear magnetic resonance measurements of the base pair kinetics^58^. We also fixed *g*=0.95 and forced the backward rate of the motor *k*^−^ to be zero. The forward rate *k*^*+*^ has been estimated from the measured maximum rewinding rate measured at low forces (*f* <15 pN). The parameters in the model allowed to vary include the following: the step size *s* and the interaction potential (Δ*G*_*a*_, *m*). As the rate of rewinding for RecG and UvsW enzymes normalized to the translocation rate followed similar dependency, both results could be reproduced with the similar set of mechanistic parameters: step size *s* of 1–2 nucleotides, stabilizing energy Δ*G*_*a*_∼4.5–6 *k*_B_*T* and interaction range *m*=2–10 bp (Fig. 3e and Supplementary Fig. S5).

## Author contributions

M.M. conducted single molecule assays, performed the analysis and prepared reversed backbone polarity DNA hairpins. M.M. and F.R. carried out OT assays. S.K.P. and P.B. prepared proteins. V.C. built the magnetic tweezers. M.M., S.K.P., P.B., F.R., S.J.B. and V.C. wrote the paper.

## Additional information

**How to cite this article:** Manosas, M. *et al*. RecG and UvsW catalyse robust DNA rewinding critical for stalled DNA replication fork rescue. *Nat. Commun.* 4:2368 doi: 10.1038/ncomms3368 (2013).

## Supplementary Material

Supplementary Information Supplementary Figures S1-S9 and Supplementary Table S1
