# Supplementary material for: RecG and UvsW catalyse robust DNA rewinding critical for stalled DNA replication fork rescue
Source: Nat Commun. 2013 Sep 9;4:2368. doi: 10.1038/ncomms3368 (PMC3778716; doi:10.1038/ncomms3368)
Supplement: Supplementary Information — Supplementary Figures S1-S9 and Supplementary Table S1 [file ncomms3368-s1.pdf]

SUPPLEMENTARY INFORMATION

For the manuscript: **RecG and UvsW catalyze robust DNA rewinding critical for stalled DNA replication fork rescue**

This supplement contains:

Supplementary Figures S1 – S9 and Supplementary Table S1

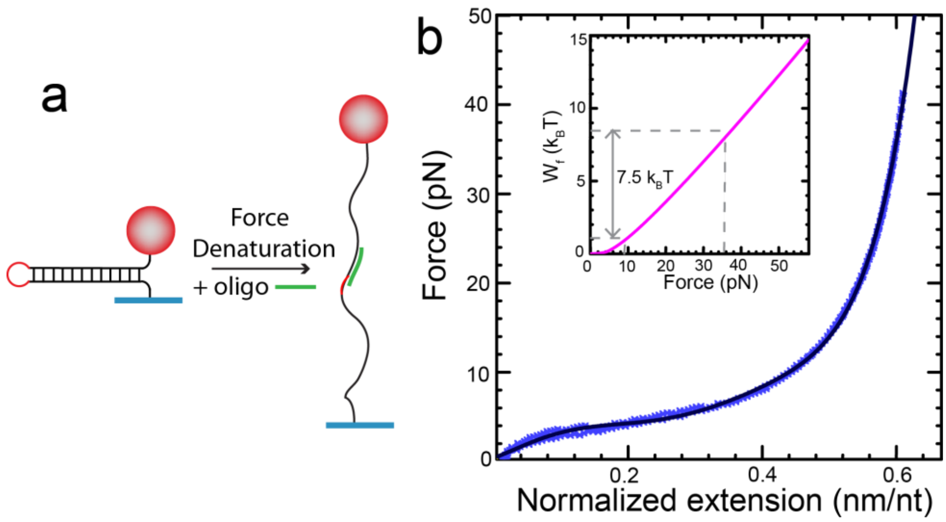

Supplementary Figure S1. Measurement of ssDNA elasticity and calibration factors. (a) The ssDNA elasticity was measured using the hairpin substrate in the presence of a 20-mer oligonucleotide complementary to the loop region of the hairpin with either optical or magnetic tweezers. The hairpin was mechanically opened by applying 18 pN in the presence of 200 nM oligonucleotide allowing the oligonucleotide to hybridize. The hybridization of the oligonucleotide generated a large (several k_BT) kinetic barrier to hairpin rewinding permitting the force-extension curve for ssDNA to be measured. (b) Experimental trace force-extension curve for the denatured 1200 bp hairpin obtained from optical tweezers experiments. The total extension has been divided by the number of nucleotides (2400 nt) of the ssDNA strand, leading to the extension x_{nt} (f) for a single nucleotide at a given force f. The continuous line is a polynomial fit to the data. Inset: Stretching work W_f required in order to bring together the two nucleotides at the junction in k_BT computed as the area under the extension-force curve. Considering the minimal free energy of formation of a base pair at the experimental conditions to be around 1 k_BT (estimated average energy of a AT base pair using Mfold[48]), W_{max} results in 7.5 k_BT.

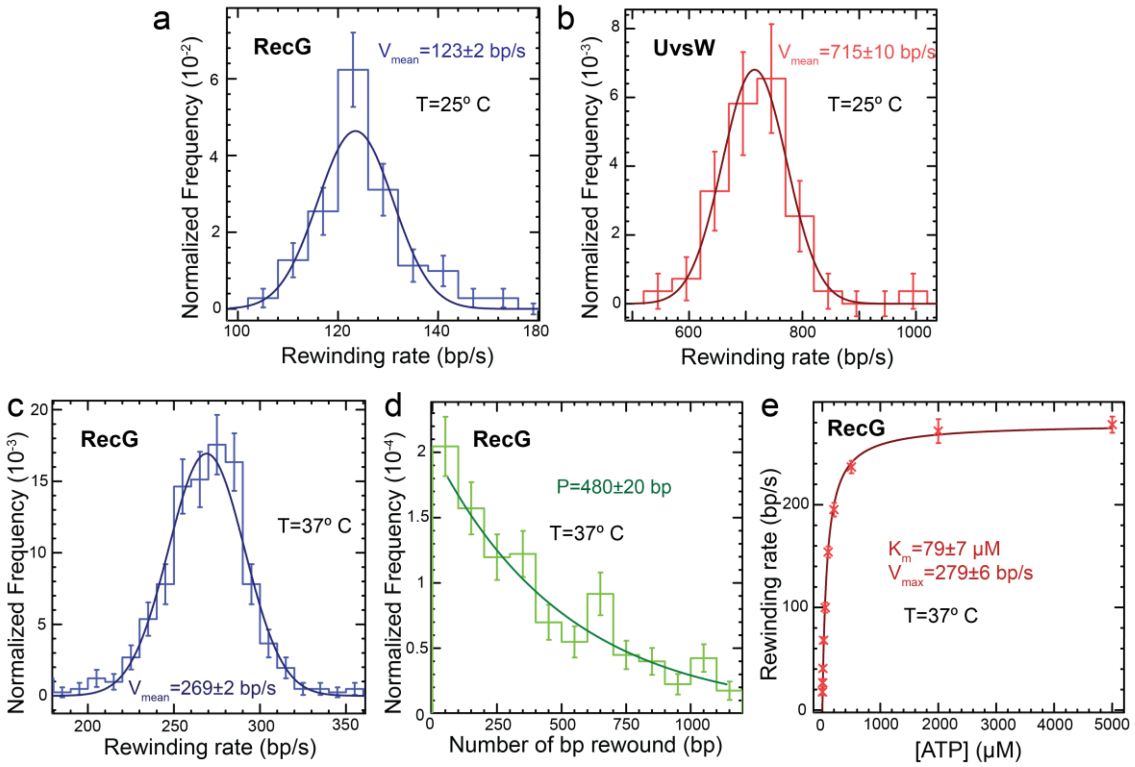

Supplementary Figure S2. Rewinding rate, processivity and ATP dependence. (a,b) Distribution of rewinding rates dZe/dt with a Gaussian fit (continuous line) for RecG (a, number *n* of experimental traces analyzed=138) and UvsW (b, *n*=121) measured at 25°C and 17 pN. Error bars are inversely proportional to the square root of the number of points for each bin. (c,d) The distribution of rewinding rates dZe/dt (c, *n*=522) with a Gaussian fit (continuous line) and the distribution of number of bases rewound (d, *n*=401) with an exponential fit (continuous line) for RecG at 37 °C and 15 pN. The exponential fit is used to estimate the enzyme processivity P. Error bars are inversely proportional to the square root of the number of points for each bin. Our results for UvsW at 37 °C leaded to mean rewinding rate and processivity of 1200 bp/s and 9 Kbp repectively[17]. (e) RecG mean rewinding rate as a function of the ATP concentration measured in the MT rewinding assays with the H_{GCapex} partially denatured hairpin at 37 °C and 15 pN of applied force. Error bars are SEM. Fitting the data to a hyperbolic saturation equation (continuous line) $r = \frac{k_{cat}[ATP]}{K_M+[ATP]}$, taking into account the error in data, a K_M and k_{cat} of 79±7 µM and 279±6 bp/s, were obtained with $\chi 2$ =8 (*n* from 420 to 125 depending on the conditions). The same ATP analysis for UvsW resulted in K_M and k_{cat} of 57 µM and 1280 bp/s[17].

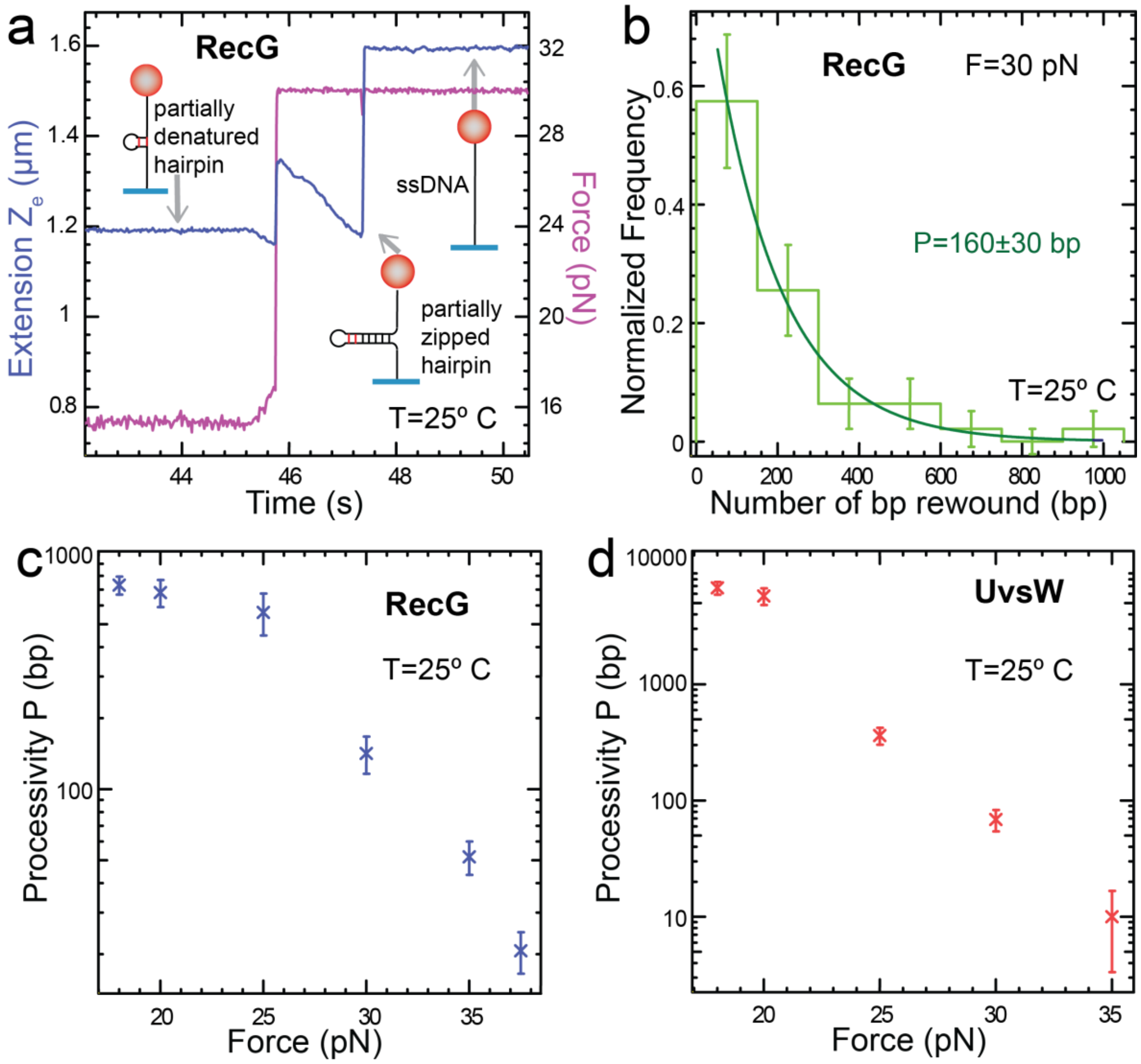

Supplementary Figure S3. RecG and UvsW processivities depend strongly on the applied force (a) Experimental trace corresponding at a force-jump OT experiment, in which starting with the partially unzipped hairpin at 16 pN, the force is increased rapidly and maintained constant to 30 pN as soon as RecG has started the annealing reaction. The measured molecular extension can be converted into the number of base pairs rewound by UvsW or RecG using a calibration factor determined from the elastic properties of ssDNA (Supplementary Fig. S1). (b) The distribution of the number of bases rewound by RecG (number *n* of experimental traces analyzed=47) at 30 pN and 25º C with an exponential fit (continuous line). The exponential fit is used to estimate the enzyme processivity P (163 bp at 30 pN). Error bars are inversely proportional to the square root of the number of points for each bin. (c,d) Processivity P as a function of the applied force for RecG (c, *n* from 74 to 37 depending on the conditions) and UvsW (c, *n* from 62 to 18 depending on the conditions). Error bars are SEM.

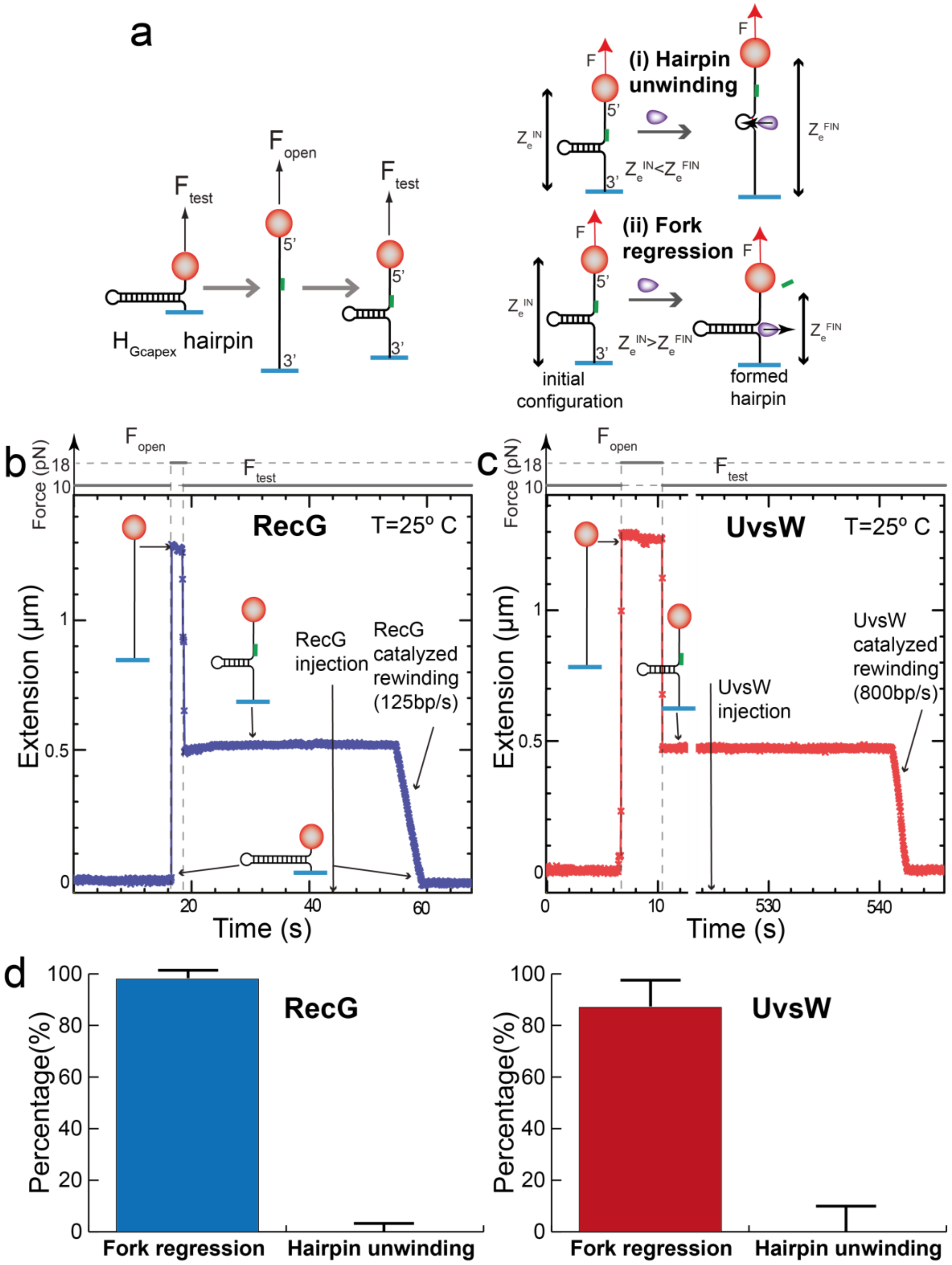

Supplementary Figure S4. RecG and UvsW simultaneous unwinding and rewinding activities to resolve branched DNA structures. (a) Schematics of construction of the branched substrate generated *in situ* in the reaction chamber. By mechanically

denaturing the H_{GCapex} hairpin and flowing a 90-mer oligonucleotide complementary to a central region of the hairpin into the chamber, a shorter hairpin was generated with long ssDNA tails containing a short dsDNA region at the junction between the dsDNA hairpin and the 5′ tail. This substrate was then used to test the ability of RecG to resolve branched structures and to measure the rewinding rate at forces below the refolding force (15 pN). Note that, a priori, two kinds of activities could be detected in such branched substrate: (i) hairpin unwinding or (ii) fork regression. (b,c) Experimental traces showing the RecG and UvsW fork regression and rewinding activity on the partially denatured hairpin with an oligonucleotide hybridized on the lagging strand at 25° C. The force was first increased to 18 pN to denature the hairpin and next relaxed to 10 pN to generate the partially denatured hairpin. RecG (b) or UvsW (c) rewound the full hairpin by expelling the hybridized oligonucleotide. The experiment was repeated at different forces F_{test} in order to compute the rewinding rate of RecG and UvsW as a function of force (light green points in Fig. 3a, 3b in the main text). (d) Percentage of experiments showing fork regression and hairpin unwinding catalyzed by RecG (number *n* of experimental traces analyzed=431) and UvsW (*n*=67). Error bars are inversely proportional to the square root of the number of experiments.

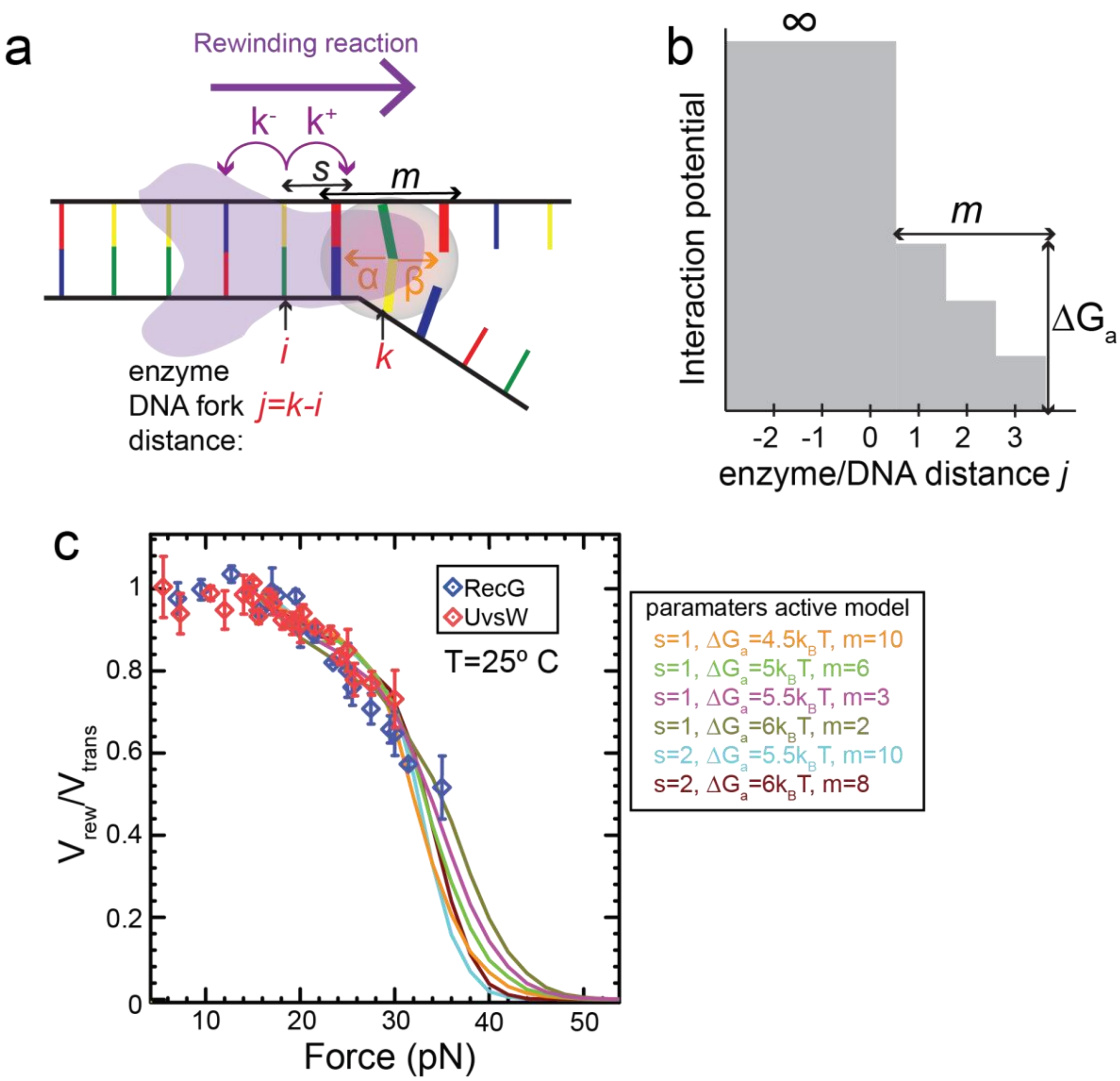

Supplementary Figure S5. Model for rewinding enzyme mechanism. (a) Schematics of the rewinding model based on the framework proposed by Betterton and Jülicher[34,35]. The parameters used in the model: α and β are the base pair opening and closing rates; k^+ and k^- are the enzyme forward and backward ssDNA translocation rates; *s* is the enzyme step size; and *m* is the range of the protein-DNA fork interaction potential. The position of the motor protein and the DNA fork along the DNA lattice is represented by the indexes *i* and *k*, respectively, and the distance between both is given by the index $j = k - i$. (b) Schematic representation of the interaction potential used in the simulations. For $j < 0$ the interaction potential tends to infinity inhibiting the DNA fork from opening when the enzyme is located directly at the ssDNA/dsDNA junction. For $j > 0$ we considered a staircase stabilizing potential of amplitude ΔG_a along a range of m nucleotides. (c) Rewinding rate normalized to the translocation rate (rewinding rate at low forces) as a function of the force for RecG (blue) and UvsW (red) motors. The

symbols correspond to experimental measurements (number n of experimental traces analyzed from 37 to 428 depending on the conditions), whereas the lines were obtained from Monte Carlo simulations based on the model described by Equations (1-5) with the indicated parameters and k_0 = 10^6 s^{-1}, g=0.95 and k^-=0. Error bars are SEM.

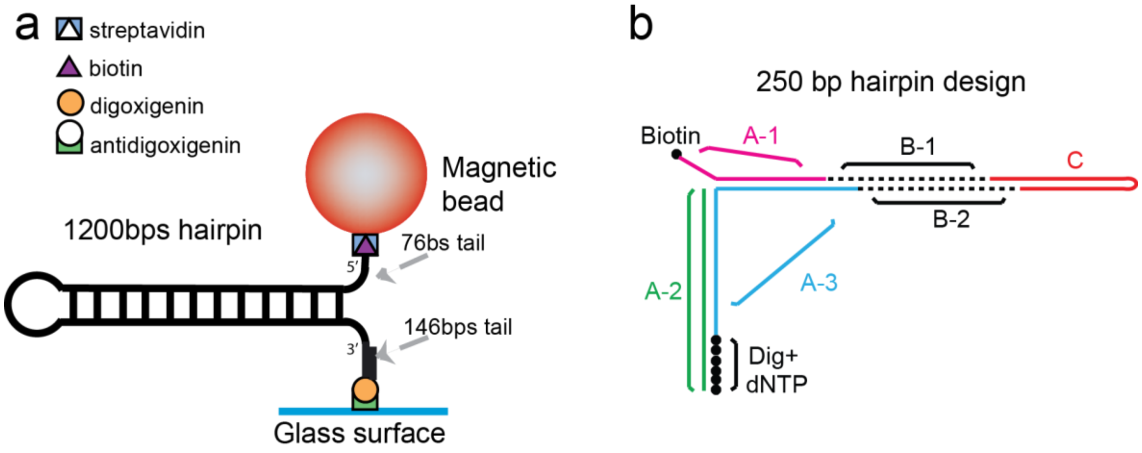

Supplementary Figure S6. DNA substrate characterization. (a) Schematic representation of the DNA hairpin substrate consisting of a 1239 bp hairpin with a loop of 4 bases, a 5′-biotinylated ssDNA tail of 76 bases, and a 3′-digoxigenin labeled dsDNA tail of 146 bp[20]. (b) Schematic representation of the 250 bp DNA hairpin substrate design. Using three different sequences for the loop oligonucleotide C (see table S1) we constructed three different hairpins: two 250 bp hairpins presenting a region with the polarity of the phosphate backbone reversed at the leading ($H_{revlead}$) or the lagging (H_{revlag}) template strand and, a 250 bp unmodified hairpin (H_0) as described in Methods.

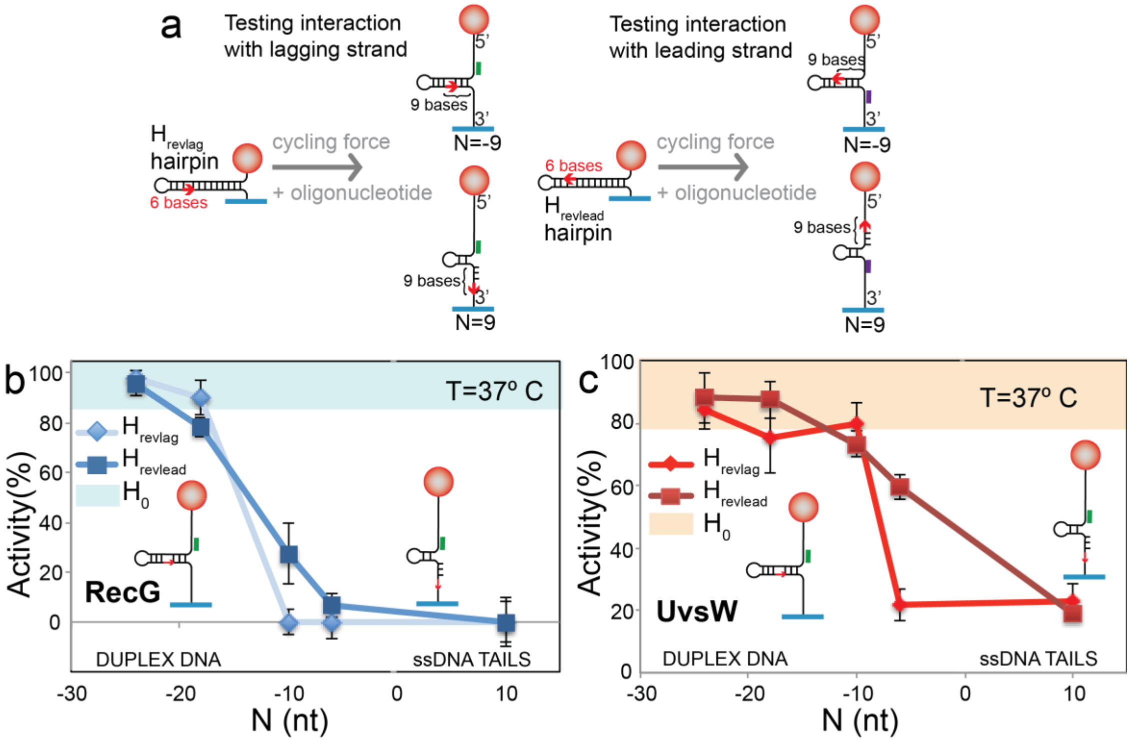

Supplementary Figure S7. Interactions with both ssDNA at the junction and the duplex DNA are required for efficient RecG and UvsW loading and initiation of rewinding reactions. (a) Schematics of the assays performed with modified hairpins with the polarity of the phosphate backbone reversed (along 6 bases) in either the leading ($H_{revlead}$) or the lagging strand (H_{revlag}). By cycling the force and employing short 40-mer oligonucleotides as a mimic of nascent strands we constructed forks *in situ* with the reversed region located at different position *N* with respect to the ssDNA/dsDNA junction. The distance in number of nucleotides *N* between the reverse block and the junction could be controlled by the choice of the oligonucleotide used; *N*>0 and *N*<0 stood for distances along the ssDNA tail and the DNA parental duplex respectively. The oligonucleotide used was chosen to be complementary to the leading strand for H_{revlag} or to the lagging strand for $H_{revlead}$ to minimize hybridization problems. (b,c) The rewinding activity, measured as the percentage of experiments showing fork regression activity, as a function of *N* for RecG (b, number *n* of experimental traces analyzed from 38 to 117 depending on the conditions) and UvsW (c, *n* from 31 to 165 depending on the conditions) and for H_{revlag}, and $H_{revlead}$ forks. Error bars are SEM. The shadow area represents the results obtained with an unmodified hairpin H_0, showing efficient rewinding activity, closed to 100 % for both RecG and UvsW. The thickness of the shadow area corresponds to variability obtained from experiments performed with different oligonucleotides, shifting the fork position along the sequence or changing N

(where N would now represent the distance in nucleotides to the unmodified initial sequence). In contrast, when the reverse block was located in either lagging or leading ssDNA tail (N>0), rewinding was inhibited. This result proved that UvsW and RecG translocation was sensitive to the polarity of both lagging and leading strands in agreement with previous studies on RecG[4,18]. Interestingly, inhibition of rewinding was also observed when placing the reverse block in the DNA parental duplex region close to the junction (*N*>-10 for UvsW and N>-20 for RecG), proving that interactions between the enzymes and the DNA duplex are necessary for binding/rewinding. For both enzymes, this inhibition was more significant when the reverse block was located in the lagging strand. These results support an scenario in which the enzyme moves along the dsDNA duplex tracking the template lagging strand towards the junction (3' to 5'), maintaining interactions with both fork tails and the duplex DNA region (Fig. 3d in the main text). The fact that inhibition in activity is also significant when the reverse block is located in the leading strand might imply that these enzymes are sensitive to the structure of the DNA duplex, which is distorted when changing the polarity of one of the strands.

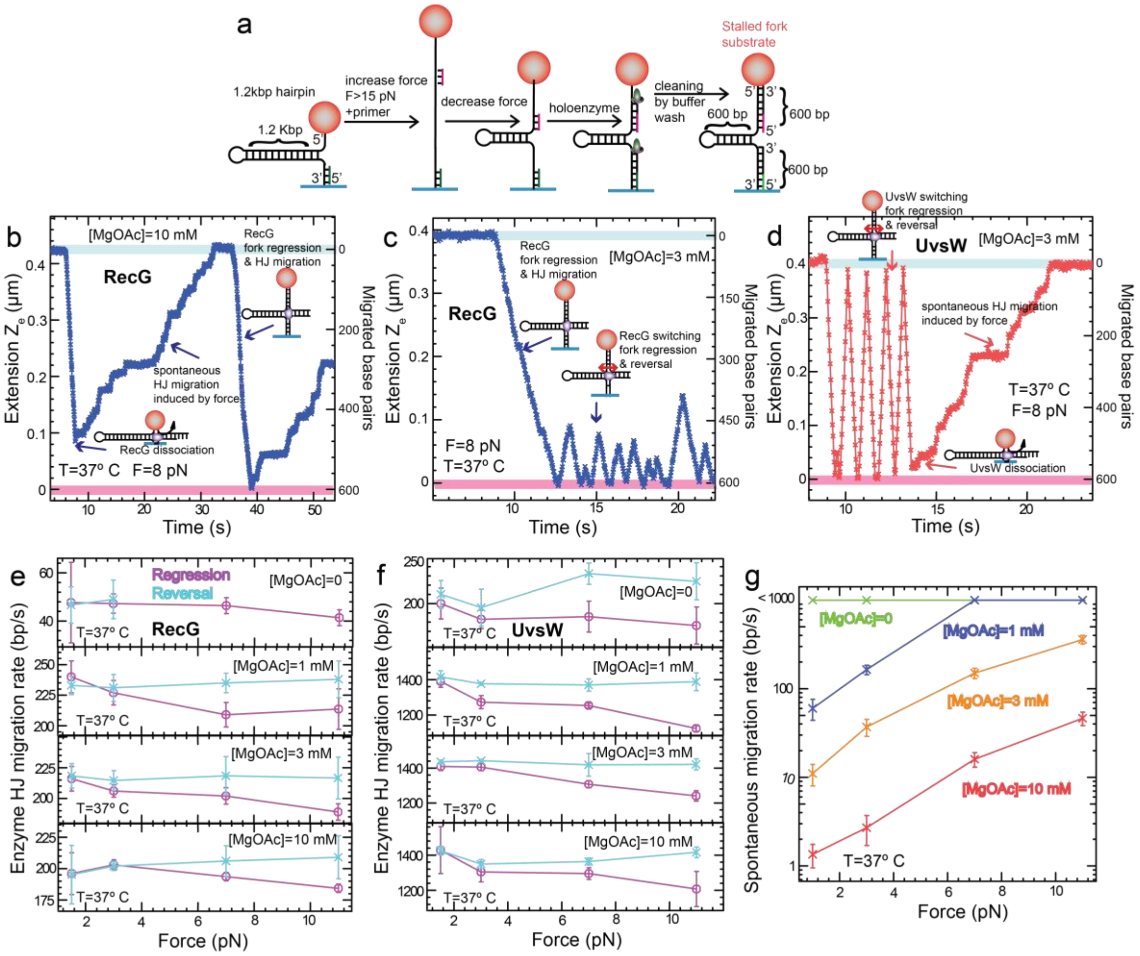

Supplementary Figure S8. Force dependence of the RecG and UvsW Holliday junction branch migration. (a) Schematics of the stalled fork substrate construction generated in situ in the reaction chamber. By mechanically denaturing the 1.2 Kbp hairpin with a built-in primer and flowing an 90-mer oligonucleotide into the chamber we built a partially denatured hairpin with primers hybridized in both leading and lagging strands. The two primers were next extended by the T4 holoenzyme complex (composed of the polymerase gp43 and the processivity clamp gp45 (14)) whose strand displacement activity could be controlled by the applied force (forces < 6 pN inhibit synthesis)[54]. Since the force applied was low (4 pN), the polymerization in the leading strand stopped upon encountering the duplex region of the hairpin and we ended up with a replication fork-like-substrate. (b,c) RecG branch migration traces performed in 10 mM (a) or 3 mM Mg^{2+} containing buffers at 37 ºC. (b). The molecular extensions corresponding to the initial stalled fork and the final fully regressed configurations are highlighted in light blue and pink respectively. (d) UvsW branch migration trace performed in buffer containing 3 mM and at 37 ºC. (e,f) RecG (d, number *n* of experimental traces analyzed from 37 to 236 depending on the conditions) and UvsW

(e, *n* from 25 to 172 depending on the conditions) mean branch migration rate during fork regression (magenta) and fork reversal (cyan) as a function of the applied force and at different Mg^{2+} ion concentration. Error bars are SEM. (g) Mean HJ spontaneous and enzyme independent branch migration rate during fork reversal as a function of the applied force and at different Mg^{2+} ion concentration (*n* from 20 to 51 depending on the conditions). Error bars are SEM. As expected for spontaneous HJ branch migration, the migration rate has a strong dependence on the applied force and Mg^{2+} ion concentration.

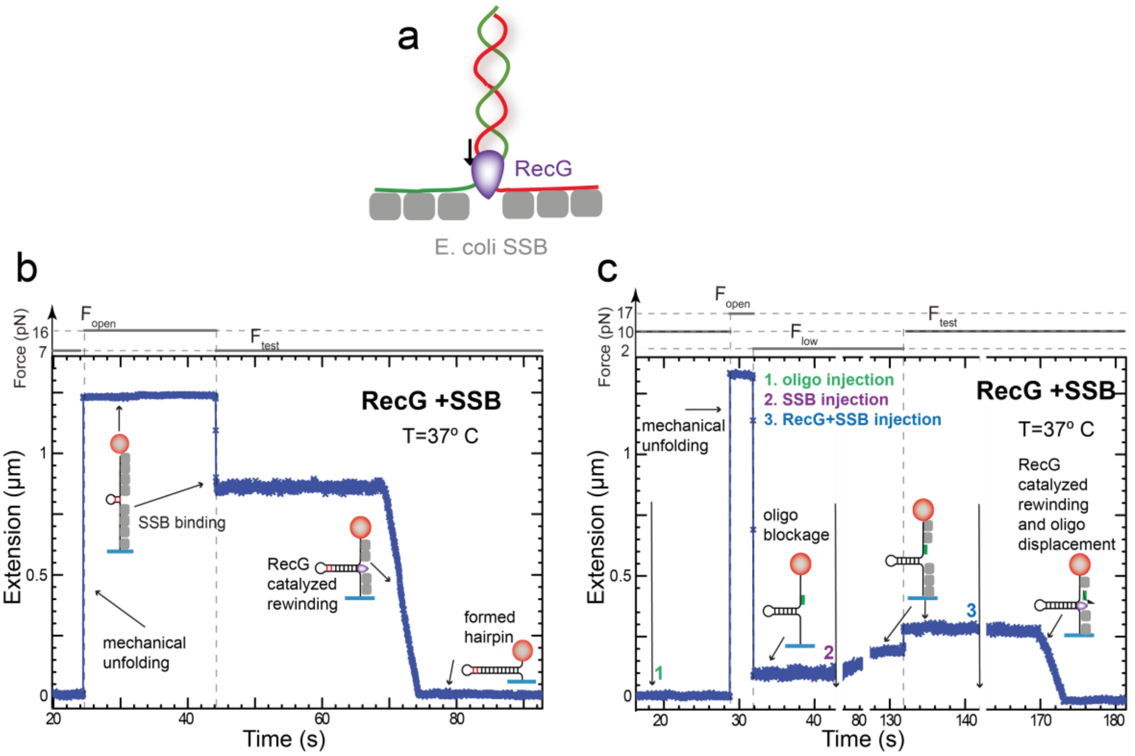

Supplementary Figure S9. RecG-catalyzed protein displacement. (a) Schematics of the assay. (b,c) Experimental traces obtained from MT hairpin rewinding assays with the H_{GCapex} in presence of *E. coli* ssDNA binding protein (SSB) at 37 ºC. In panel b, 16 pN of force was first applied generating a partially denatured hairpin with long ssDNA tails. DNA coating was then achieved by injection of 50 nM tetramer SSB. By decreasing the force to 7 pN the molecule did not refold and presented a fixed extension, a signature of a stable coating. After injection of RecG and ATP (together with SSB), rewinding events were observed. In panel c, a partially denatured hairpin was first generated by modulating the force and injecting a 90-mer oligonucleotide complementary to the central region of the hairpin (1). The force was then decreased at 2 pN and 50 nM SSB was injected (2). At this low force the coating of SSB along the ssDNA tails was detected as an increase in Z_e. The force was finally increased to 10 pN and RecG and ATP was added to the solution (3) leading to the full rewinding of the hairpin. The average rewinding rate measured from theses assays was 260 ± 30 bp/s (number *n* of experimental traces analyzed=22), close to the measured rewinding rate in absence of SSB (~280 bp/s). Similar results are obtained for UvsW displacing T4 ssDNA binding protein gp32[17].

Supplementary Table S1. Oligonucleotides (Eurogentec and Integrated DNA technology) used to construct the 250 bp DNA hairpins, as shown in Supplementary Fig. S6. Regions of reversed phosphate backbone polarity are shown in red.

**A-1**	5′-biotin-TTT TTT TTT TTT TTT TTT TTT TTT TTT TTT TTT TTT TTT TTT TTT TTT TTG GAT TCG CGG GTC TCT-3′
**A-2**	5′-AAC CGT CCT TTA CTT GTC ATG CGC TCT AAT CTC TGG GCA TCT GGC TAT GAT GTT GAT GGA ACT GAC CAA ACG TCG GTG GG-3′
**A-3**	5′-phosphorylated-AGG AAG AGA CCC GCG AAT CCC CCA CCG ACG TTT GGT CAG TT-3′
**B-1**	5′-phosphorylated- TCC TCG TGC GTG AGC GAG CGC GGT CGG TCG GTC GGT AGC GAG CGC GTG CGT GCG TGC GTG GGC TGG CTG GCT GGC TCG GTC GGT CGT GCG TGC GGT CGG TGG CTG GCT AGC GAG CGA GCG AGC GGG CTG GCT GAA GAC TT -3′
**B-2**	5′-phosphorylated- GCG AAA GTC TTC AGC CAG CCC GCT CGC TCG CTC GCT AGC CAG CCA CCG ACC GCA CGC ACG ACC GAC CGA GCC AGC CAG CCA GCC CAC GCA CGC ACG CAC GCG CTC GCT ACC GAC CGA CCG ACC GCG CTC GCT CAC GCA CG -3′
**C** **H_{revlag}**	5′-phosphorylated- TCG CGG AGC ATG CGG C*GG GAT G*CG ACG TCG GGC CGT CAG ATG CCT TTT TTG GCA TCT GAC GGC CCG ACG TCG CAT CCC GCC GCA TGC TCC -3′
**C** **$H_{revlead}$**	5′-phosphorylated- TCG CGG AGC ATG CGG CGG GAT GCG ACG TCG GGC CGT CAG ATG CCT TTT TTG GCA TCT GAC GGC CCG ACG TCG *CAT CCC* GCC GCA TGC TCC -3′
**C** **H_0**	5′-phosphorylated- TCG CGG AGC ATG CGG CGG GAT GCG ACG TCG GGC CGT CAG ATG CCT TTT TTG GCA TCT GAC GGC CCG ACG TCG CAT CCC GCC GCA TGC TCC -3′
